# Supplementary material for: The superficial tufted and mitral cell output neurons of the mouse olfactory bulb have dual roles in insulin sensing
Source: J Cell Sci. 2025 Oct 13;138(19):jcs264088. doi: 10.1242/jcs.264088 (PMC12579955; doi:10.1242/jcs.264088)
Supplement: Supplementary information [file joces-138-264088-s1.pdf]

Table S1. Comparison of the action potential properties of mitral vs. tufted cells in mouse olfactory bulb slices. Recordings were made while holding cells near rest (V_h = - 60 mV) and injecting 75 pA of current. Reported values represent the mean ± s.d. with noted sample size (N) as number of slice recordings. ns = not significantly different; **bolded** values are significantly different at criteria *p* < 0.05 with actual *p* value reported. †= Student's *t*-test, ‡= *t*-test with Welch's correction, ₸= Mann-Whitney U.

	Peak Amplitude (mV)	Time to Peak Amplitude (ms)	Antipeak Amplitude (mV)	Time to Antipeak (ms)	Half Width (FWHM) (ms)
Mitral Cells	51.1 ± 10.0	5.83 ± 0.26	-12.1 ± 2.4	11.4 ± 0.7	1.78 ± 0.36
N	11	11	11	11	11
Superficial Tufted Cells	60.8 ± 7.0	5.72 ± 0.09	-15.1 ± 2.5	9.20 ± 0.90	1.32 ± 0.16
N	11	11	11	11	11
p-value	**0.0150**	ns (0.1970)	**0.0079**	**< 0.0001**	**< 0.0001**
Test used	†	†	₸	†	₸

	Max Rise Slope (mV/ms)	Time to Max Rise Slope (ms)	Max Decay Slope (mV/ms)	Time to Max Decay Slope (ms)	Rise Time to Half Amplitude (ms)
Mitral Cells	88.4 ± 35.2	5.11 ± 0.17	-44.3 ± 13.2	0.866 ± 0.167	5.09 ± 0.11
n	15	11	15	15	11
Superficial Tufted Cells	117.0 ± 44.0	5.06 ± 0.17	-79.5 ± 20.4	0.612 ± 0.105	5.11 ± 0.05
n	20	11	20	20	11
p-value	**0.0493**	ns (0.8338)	**< 0.0001**	**< 0.0001**	ns (0.7969)
Test used	†	₸	‡	†	₸

	Decay Time to Half Amplitude (ms)	Rise Slope 10% to 90% (mV/ms)	Rise Time 10% to 90% (ms)	Decay Slope 90% to 10% (mV/ms)	Decay Time 90% to 10% (ms)
Mitral Cells	6.87 ± 0.38	58.8 ± 24.1	1.04 ± 0.82	-31.3 ± 8.2	1.41 ± 0.21
n	11	11	11	11	11
Superficial Tufted Cells	6.43 ± 0.16	74.1 ± 18.3	0.784 ± 0.151	-56.7 ± 11.2	0.911 ± 0.107
n	11	11	11	11	11
p-value	**0.0021**	ns (0.1087)	ns (0.3192)	**< 0.0001**	**< 0.0001**
Test used	†	‡	†	‡	†

Journal of Cell Science • Supplementary information

Table S2. Spike bursting properties in superficial tufted cells (sTCs) compared between wildtype (WT) and Kv1.3 -/- mice. Recordings were made while holding cells near rest (V_h = -60 mV) and injecting 75 pA of current. Data were computed across a 50 s recording period (10, 5s sweeps). ns = not significantly different; **bolded** values are significantly different at criteria *p* < 0.05 with actual *p* value reported. †= Student's *t*-test, ‡= *t*-test with Welch's correction, ₸= Mann-Whitney U.

	Bursts per 50 s (n)	Spikes per Burst (n)	Burst Duration (ms)	Intraburst Interval (ms)	Intraburst Frequency (Hz)
Kv1.3 +/+ sTCs	24.8 ± 10.0	7.6 ± 3.1	414.6 ± 209.5	65.1 ± 12.9	17.0 ± 5.6
n	11	11	11	11	11
Kv1.3 -/- sTCs	55.0 ± 9.6	6.0 ± 1.4	215.0 ± 82.4	45.6 ± 11.7	26.1 ± 8.7
n	4	4	4	4	4
p-value	**0.0003**	ns (0.2362)	**0.0305**	**0.0291**	**0.0048**
Test used	†	‡	‡	†	₸

	Pause Duration (ms)	Latency to First Spike (ms)	AP Frequency (Hz)	Event Frequency (Hz)	% Bursting Cells
Kv1.3 +/+ sTCs	729.8 ± 354.1	1221.6 ± 689.4	4.8 ± 1.7	7.6 ± 2.6	
n	11	11	11	11	
Kv1.3 -/- sTCs	655.3 ± 210.4	419.5 ± 298.7	7.1 ± 2.2	7.6 ± 2.5	
n	4	5	5	5	
p-value	ns (0.7183)	**0.0090**	**0.0475**	ns (0.7427)	
Test used	†	‡	†	₸	

Journal of Cell Science • Supplementary information
